# Light-Excited Ag-Doped TiO_2_−CoFe_2_O_4_ Heterojunction Applied to Toluene Gas Detection

**DOI:** 10.3390/nano11123261

**Published:** 2021-11-30

**Authors:** Wenhao Wang, Lu Zhang, Yanli Kang, Feng Yu

**Affiliations:** 1 Key Laboratory for Green Processing of Chemical Engineering of Xinjiang Bingtuan, School of Chemistry and Chemical Engineering, Shihezi University, Shihezi 832003, China; 18299083557@163.com (W.W.); 13040547128@163.com (Y.K.); 2 Harbin Institute of Technology, School of Science, Shenzhen 518055, China; 18B925124@stu.hit.edu.cn; 3 Bingtuan Industrial Technology Research Institute, Shihezi University, Shihezi 832003, China; 4 Carbon Neutralization and Environmental Catalytic Technology Laboratory, Shihezi University, Shihezi 832003, China

**Keywords:** toluene, gas sensor, noble metal, heterojunction, gas sensor, light

## Abstract

(1) Background: Toluene gas is widely used in indoor decoration and industrial production, and it not only pollutes the environment but also poses serious health risks. (2) Methods: In this work, TiO_2_−CoFe_2_O_4_−Ag quaternary composite gas-sensing material was prepared using a hydrothermal method to detect toluene. (3) Results: The recombination of electron–hole pairs was suppressed, and the light absorption range was expanded after constructing a heterojunction and doping with Ag, according to ultraviolet–visible (UV–vis) diffuse reflectance spectra and photoluminescence spectroscopy. Moreover, in the detection range of toluene gas (3 ppm–50 ppm), the response value of TiO_2_−CoFe_2_O_4_−Ag increased from 2 to 15, which was much higher than that of TiO_2_−Ag (1.7) and CoFe_2_O_4_−Ag (1.7). In addition, the working temperature was reduced from 360 °C to 263 °C. Furthermore, its response/recovery time was 40 s/51 s, its limit of detection was as low as 10 ppb, and its response value to toluene gas was 3–7 times greater than that of other interfering gases under the same test conditions. In addition, the response value to 5 ppm toluene was increased from 3 to 5.5 with the UV wavelength of 395 nm–405 nm. (4) Conclusions: This is primarily due to charge flow caused by heterojunction construction, as well as metal sensitization and chemical sensitization of novel metal doping. This work is a good starting point for improving gas-sensing capabilities for the detection of toluene gas.

## 1. Introduction

With the development of the economy and the substantial improvement at industrial level, there are a growing number of toxic and harmful gases in the air, which pollute the environment and also seriously endanger human health [1,2,3]. Toluene gas, for example, produced during indoor decoration, has been linked to vision impairment; asthma; nasopharyngeal cancer; and other diseases and health events, including pregnancy loss [4,5,6,7]. In addition, when people are exposed to high concentrations of toluene vapor (200 ppm–500 ppm), various symptoms, such as headache, nausea, muscle cramps, and dizziness, may occur. When the human body is exposed to a too high concentration of toluene gas, the brain is permanently poisoned and may even die [8]. As a result, an efficient and sensitive gas sensor for the detection of toluene is required.

The gas sensors made of semiconductor metal oxide gas-sensitive materials have low cost, high sensitivity, and simple operation [4], which are easy to integrate into mobile phones and other miniaturized devices [5]. Common gas-sensitive materials include SnO_2_ [6,7], ZnO [8,9], TiO_2_ [10,11], WO_3_ [12,13], Fe_2_O_3_ [14], In_2_O_3_ [15,16], and Co_3_O_4_ [17,18]. TiO_2_ is one of them, and it is widely used in gas detection because of its advantages, such as good stability, non-toxicity, and recyclability [19,20]. However, pure TiO_2_-based gas sensors have high resistance, poor selectivity, and low response strength, limiting their wide application. As we all know, it can be modified by some methods, such as doping [21], constructing heterojunctions, defect regulation, and controlling morphology [22,23,24,25,26]. The use of heterostructures can improve the material’s catalytic activity and adsorption capacity, thereby enhancing sensor signal reception and transduction. Li et al. [27] prepared TiO_2_@SnO_2_ hollow nanospheres using a hydrothermal method with the aid of templates. When compared to pure hollow TiO_2_ nanospheres, hollow TiO_2_@SnO_2_ heterojunction nanospheres performed better in terms of formaldehyde sensing under ultraviolet (UV) irradiation at room temperature. The response/recovery time was shortened from 52 s/164 s to 20 s/56 s. This phenomenon indicated that the heterostructure could improve the electron transfer efficiency and enhance the gas sensitivity.

Furthermore, element doping can affect the surface defects and electrical properties of the sensing material, which promotes electron transfer. Simultaneously, element doping can alter the material bandgap, improving gas sensitivity. In particular, doping platinum, gold, palladium, silver, and other precious metals with high catalytic activity and low Fermi levels will trigger electronic sensitization and chemical sensitization effects, thus effectively improving the material’s gas sensitivity [28]. Pan et al. [29] formed Pd-doped TiO_2_ nanofiber membranes with varying doping amounts using rotating surface flame stabilization technology (FSRS). It was discovered that noble metal doping improved the response intensity of the TiO_2_-based sensor to CO, which also greatly reduced its response/recovery time to NH_3_.

In this work, TiO_2_−CoFe_2_O_4_−Ag quaternary composite gas-sensitive material is synthesized with a hydrothermal method to detect toluene gas. The method for constructing heterojunctions and doping noble metals greatly improves the detection ability of TiO_2_-based gas-sensitive materials for toluene gas. It is discovered that the recombination of photogenerated electron–hole pairs is inhibited, and absorbance increases significantly, particularly in the UV region. Moreover, it can be seen that the working temperature is greatly reduced; the response value is increased compared with TiO_2_−Ag and CoFe_2_O_4_−Ag. At the same time, its gas sensitivity response value is further increased with UV irradiation.

## 2. Materials and Methods

### 2.1. Preparation of Materials

First, 5.95 g of cobalt chloride hexahydrate and 13.51 g of ferric chloride hexahydrate were placed into 100 mL of ethylene glycol solution with ammonium acetate. The solution was then stirred at 300 rpm at room temperature for one night before being aged for another. Following that, the suspension was hydrothermally treated for 24 h at 180 °C. After it cooled down, it was centrifuged at a speed of 8000 r/min and cleaned with 1 L of deionized water and anhydrous ethanol alternately. Finally, the product was dried at 60 °C for 12 h before being prepared with CoFe_2_O_4_. TiO_2_−CoFe_2_O_4_ was prepared in the same way as CoFe_2_O_4_, except that a suspension containing 2.00 g of titanium dioxide was added before the hydrothermal treatment. TiO_2_−Ag, CoFe_2_O_4_−Ag, and TiO_2_−CoFe_2_O_4_−Ag were all prepared by dipping with silver nitrate solution as a silver source; the doping amount of silver in the three materials was 1% wt.

The concentration of ethylene glycol is 98%. All other materials and reagents are analytical reagents.

### 2.2. Material Characterization

The crystal structures of the samples were recorded using a powder X-ray diffraction method (XRD, BRUCKERD8 ADVANCE, Karlsruhe, Germany) with Co Kα radiation at a scan rate of 8°/min. Scanning electron microscope (SEM, SU8020, Hitachi Corporation, Tokyo, Japan), transmission electron microscope (TEM), and high-resolution TEM (HRTEM) with FEI Tecnai G2 F30 were used to characterize the morphology and microstructure (FEI Corporation, Hillsboro, OR, USA). The specific area and pore diameter distributions were estimated using the Brunauer–Emmett–Teller equation and the Barrett–Joyner–Halenda method based on N_2_ adsorption isotherms (ASAP 2460, Micromeritics Instruments Corporation, NJ, USA). Thermo scalable 250 Xi equipment was used for X-ray photoelectron spectroscopy (XPS) measurements (FEI Corporation, Hillsboro, OR, USA). The ultraviolet–visible diffuse reflectance spectra (UV–vis DRS) of the samples were measured using the UV-3600 instrument (SHIMADZU Corporation, Kyoto, Japan). An FLS1000/FS5 fluorescence spectrometer (excitation wavelength = 350 nm) was employed to obtain the photoluminescence (PL) spectra (Edinburgh Company, Edinburgh, UK), which were used to analyze the recombination behavior of photoinduced carriers.

### 2.3. Sensing Performance Evaluation

The CGS-8 intelligent gas sensor system was used to evaluate sensing performance (Beijing Elite Co., Ltd., Beijing, China). Before the performance test, the gas-sensitive material was mixed evenly with ethanol and applied to the surface of the ceramic tube, which was welded to the hexapod base. Then, it was aged at 150 °C for 48 h after standing for a while. The light excitation performance test was carried out by irradiating about 3 cm above the gas sensor. During the performance test, the relative humidity (RH) was around 12%. The sensors’ responses were calculated using the following equation:

Response = Ra/Rg(1)

where Ra is the resistance of the sensor in air, and Rg is the electrical resistance for the sensor in the tested gas.

## 3. Results and Discussion

### 3.1. Structural and Morphological Characterization

**Figure 1:**
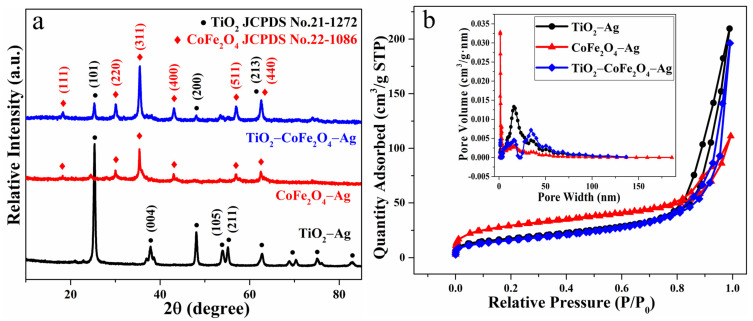
(**a**) X-ray diffraction (XRD) patterns and (**b**) N_2_ adsorption–desorption isotherms, with pore size distributions (inset) of TiO_2_−Ag, CoFe_2_O_4_−Ag, and TiO_2_−CoFe_2_O_4_−Ag.

From the XRD test results in Figure 1a, the typical anatase peak pattern of TiO_2_ can be seen; the peaks appearing at 2*θ* = 25.347°, 37.861°, 48.073°, 53.922°, 55.164°, and 62.788° correspond with (101), (004), (200), (105), (211), and (213) and other crystal planes of TiO_2_, respectively (JCPDS No.21-1272) [30,31]. When compared to other peaks, the intensity of the peak at 25.347° is the highest, indicating that the corresponding (101) crystal plane is the most exposed. The peak state compares to that reported in the literature, confirming the presence of titanium dioxide in the crystal phase of anatase [32,33]. The anatase nanocrystals have a higher specific surface area and a greater concentration of oxygen vacancies, so there may be more active centers and higher charge separation efficiency. Furthermore, because the bandgap of anatase is relatively large, its redox ability is slightly higher than that of rutile. As a result, anatase has improved photocatalytic performance and is the ideal TiO_2_ nanocrystalline phase for gas-sensitive reactions [34,35]. Compared with TiO_2_−Ag, the peak intensity of TiO_2_−CoFe_2_O_4_−Ag becomes weaker, which indicates that titanium dioxide nanoparticles may be affected after being compounded with a cobalt ferrite. Similar reports have been reported in the previous literature [36].

The peaks of CoFe_2_O_4_ at 2*θ* = 18.231°, 30.016°, 35.376°, 43.001°, 53.347°, 56.878°, and 62.483° correspond with (111), (220), (311), (400), (422), (511), and (440) of CoFe_2_O_4_ crystal planes (JCPDS No.22-1086) [33,37]. The peak state is consistent with what has been reported in the literature [35,38]. The intensity of the peak at 35.376° is greater than that of the other peaks, indicating that the corresponding (311) crystal plane is more exposed. Due to too little Ag doping, there is no obvious characteristic peak of Ag in the spectrum. Furthermore, there are no impurity peaks in all three materials, which shows that the synthesized samples are relatively pure.

Figure 1b shows that the pore diameters of the three types of gas-sensitive materials are concentrated in the 10 nm–40 nm range, with mesopores being the most common, followed by a few micropores. It can be seen in Table 1 that the specific surface area, pore volume, and pore size of the TiO_2_−CoFe_2_O_4_−Ag gas-sensing material are all closer to those of TiO_2_−Ag after recombination but slightly reduced. While these characteristics are related to gas-sensing performance, they are not the only deciding factors. The gas sensitivity of a material is also affected by its internal structure and surface defects [39].

**Figure 2:**
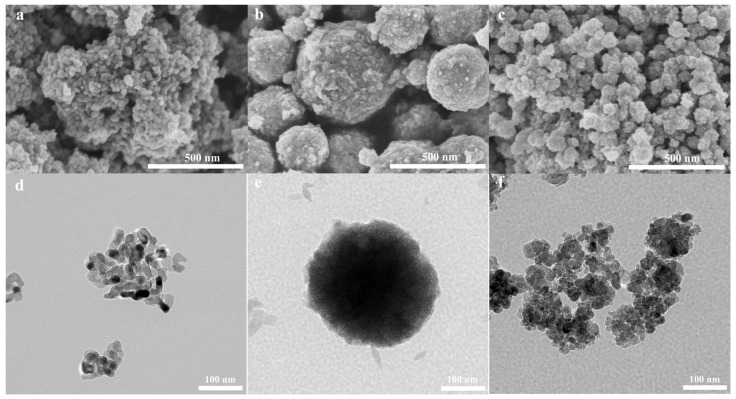
Scanning electron microscope (SEM) and transmission electron microscope (TEM) morphologies of TiO_2_−Ag (**a**,**d**), CoFe_2_O_4_−Ag (**b**,**e**), and TiO_2_−CoFe_2_O_4_−Ag (**c**,**f**).

The SEM and TEM images in Figure 2a–f show that TiO_2_−Ag is made up of small rice-shaped particles that stack together to form a coral-like structure. CoFe_2_O_4_−Ag, on the other hand, has a nearly spherical structure with a regular shape. The diameter varies from about 300 nm to 700 nm. The size of TiO_2_−CoFe_2_O_4_−Ag is bigger than that of TiO_2_−Ag and smaller than that of CoFe_2_O_4_−Ag. TiO_2_−CoFe_2_O_4_−Ag is in the shape of a sphere, which stacks together, and some individual boundaries blend. The structure has numerous holes and voids to ensure that the gas-sensitive material fully contacts and reacts with the air and target gas, improving gas-sensitive performance and shortening reaction time. At the same time, it can be seen that there is some debris on the surface of the three materials, which may be the Ag substance.

**Figure 3:**
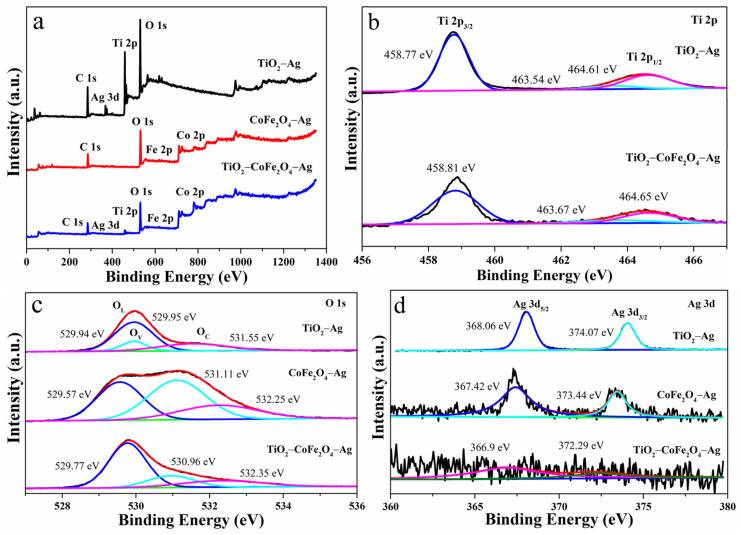
X-ray photoelectron spectroscopy (XPS) spectra: (**a**) total spectrum, (**b**) Ti 2p, (**c**) O 1s, and (**d**) Ag 3d.

As the binding energy increases from low to high, the XPS total spectrum (Figure 3a) shows six features of C, Ag, Ti, O, Fe, and Co. Apart from the C element derived from the test process, no other impurity elements are present, indicating that the gas-sensitive material is pure and pollution free.

In Figure 3b, the binding energy of the two peaks of Ti 2p from low to high is attributed to Ti 2p_3/2_ and Ti 2p_1/2_, respectively, and the difference between the two peaks is about 5.5 eV, indicating that the material contains TiO_2_. The Ti 2p_1/2_ peak can be divided into two peaks, 463.54/463.67 eV and 464.61/464.65 eV, indicating the presence of Ti^3+^. To maintain the charge balance, there must be oxygen vacancies, which can improve the gas sensitivity response.

Figure 3c shows the O 1s peaks that can be attributed to lattice oxygen (O_L_), defect oxygen (O_V_), and adsorbed oxygen (O_C_). Among them, the binding energy of O_L_ is 529.94/529.57/529.77 eV, which is primarily derived from the oxygen within the TiO_2_ and CoFe_2_O_4_ lattices. The binding energy of O_V_ is located at 529.95/531.11/530.96 eV, indicating that there are some oxygen defects, which mainly exist in the form of oxygen vacancies. This corresponds to the result of the Ti 2p elemental analysis. The binding energy of O_C_ is 531.55/532.25/532.35 eV, which comes from the chemically adsorbed and dissociated oxygen elements, indicating that there is a certain amount of oxygen anions in different states ((O_2_^−^ (ads), O^−^ (ads), and O^2−^ (ads)), which are helpful for the response of metal oxide semiconductor gas-sensitive materials.

The presence of Ag in Figure 3d indicates that the element is doped successfully. The two peaks are Ag 3d_5/2_ and Ag 3d_3/2_, and their binding energies are 368.06/367.42/366.9 eV and 374.07/373.44/372.29 eV. The binding energy difference is approximately 6 eV, indicating that the deposited silver nanoparticles are metallic silver [40,41]. At the same time, because the element Ag occupies a relatively small proportion, the peaks are not obvious, and the peak intensity is low.

**Figure 4:**
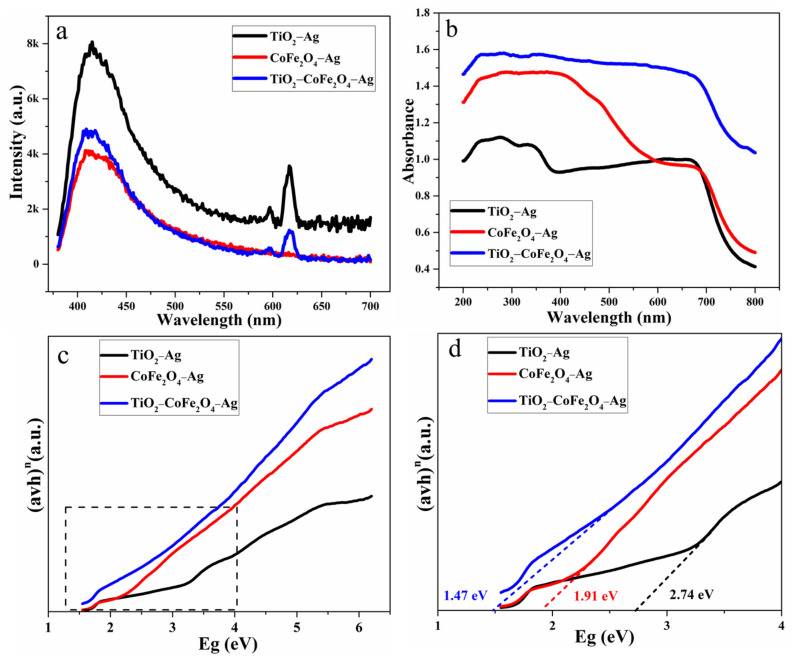
(**a**) Photoluminescence (PL) spectra; (**b**) ultraviolet–visible diffuse reflectance spectra (UV–vis DRS); and (**c**,**d**) optical band gap of TiO_2_−Ag, CoFe_2_O_4_−Ag, and TiO_2_−CoFe_2_O_4_−Ag.

It can be seen that the TiO_2_−CoFe_2_O_4_−Ag quaternary composite material and TiO_2_−Ag have the same peaking law in Figure 4a. Peaks between 400 nm and 450 nm are near-band edge peaks that are typically accompanied by the generation of electron–hole pairs. When an electron is excited to transition to the conduction band, the holes in the valence band interact and self-annihilate, releasing the energy difference between the highest occupied molecular orbital energy state in the valence band and the lowest unoccupied molecular orbital energy state in the valence band minus the excitons’ binding energy [42]. The peak between 600 nm and 650 nm corresponds to the red emission band, indicating that there are a large number of defects in the material, which are conducive to the improvement of its gas-sensing performance. Among the three materials, the peak intensity of CoFe_2_O_4_−Ag and TiO_2_−CoFe_2_O_4_−Ag is significantly lower than that of TiO_2_−Ag, indicating that the recombination of photogenerated electron–hole pairs is significantly suppressed and that more carriers can be generated under light conditions. It is helpful to improve the conductivity of the material and reduce the operating temperature.

A large portion of solar radiation is visible light (43%) and infrared (52%), with only 5% falling into the UV range. Only colorless and white substances can absorb energy in the UV range. Pure TiO_2_ is a white crystalline solid that only absorbs UV energy. This is related to the intrinsic bandgap absorption of typical anatase TiO_2_, which is caused by the transition of electrons from the valence band to the conduction band [43]. Moreover, by modification through a series of methods, such as doping and constructing heterojunctions, the light absorption range can be expanded to include visible light [44]. Figure 4b shows that its light absorption range is 200 nm–700 nm, which covers the visible, UV, and infrared regions, indicating that the addition of the noble metal Ag improves its utilization ability to different wavelengths of light. This may cause different wavelengths of light to enhance the gas sensitivity of the material.

The absorption in the visible region of the spectrum is low, but the absorption in the UV region is high, indicating that it preferentially absorbs UV light. At the same time, due to the effect of local surface plasmon resonance, the absorption of TiO_2_−Ag in the visible region (400 nm–700 nm) will be increased accordingly [45,46]. Furthermore, the absorption light intensity of TiO_2_−CoFe_2_O_4_−Ag is significantly greater than that of TiO_2_−Ag and CoFe_2_O_4_−Ag, indicating that it has a higher light utilization rate. When a gas-sensitive reaction occurs, the light enhancement may be more pronounced.

The bandgap energy is calculated using the Kubelka–Munk (KM) method:

α*hυ* = A (*hν* − Eg)^n^ (2)

where A is an absorbance constant, *hν* is the energy of discrete photons, and α is the absorption coefficient, which can be calculated from the diffuse reflectance data using the KM method. The bandgap energy is denoted by the symbol Eg. n is 0.5 because titanium dioxide and other materials have an indirect bandgap. Finally, the bandgap value can be obtained by extrapolating the intersection of (α*hυ*)^n^ and photon energy (*hυ*) [42,47].

It can be seen in Figure 4c,d that the bandgap of TiO_2_−Ag is about 2.74 eV, the bandgap of CoFe_2_O_4_−Ag is 1.91 eV, and the bandgap of TiO_2_−CoFe_2_O_4_−Ag is about 1.47 eV. The bandgap is reduced by constructing heterojunctions and doping Ag. As a result, electrons transition more easily, and gas sensitivity may be improved.

### 3.2. Gas-Sensing Characteristics

**Figure 5:**
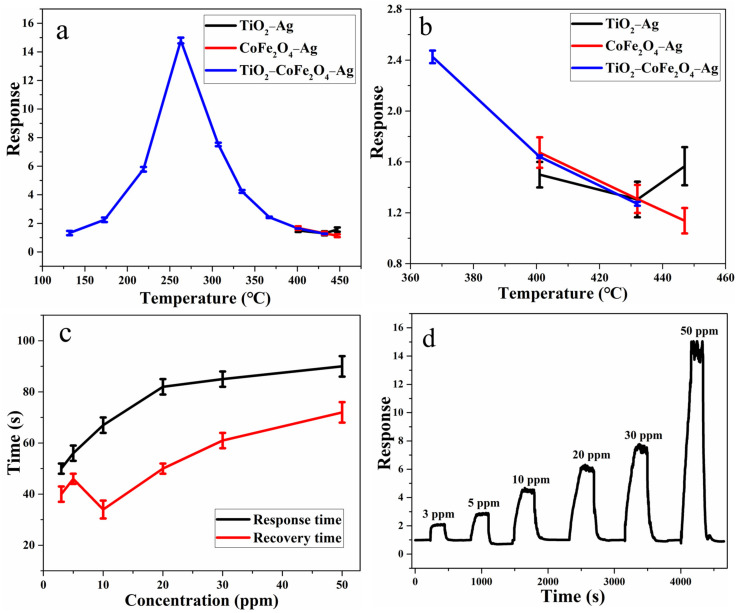
(**a**) Gas-sensitive response test to toluene at different temperatures (n = 3), (**b**) gas-sensitive response test to toluene at 360–455 °C (n = 3), (**c**) response/recovery time (n = 3), and (**d**) dynamic response of TiO_2_−CoFe_2_O_4_−Ag to toluene gas at 263 °C.

The working temperature has a direct effect on the surface activity state of the gas-sensitive material and the diffusion rate of gas molecules on the surface of the gas-sensitive material, which in turn affects the sensor device’s sensitivity. Therefore, the influence of the working temperature on the gas-sensing performance is tested first. It can be seen from Figure 5a, b that the response value of TiO_2_−CoFe_2_O_4_−Ag to 50 ppm toluene presents the trend of first increasing and then decreasing with an increase in working temperature. It is hypothesized that this is because the energy required for the reaction of toluene with chemically adsorbed oxygen on the surface of gas-sensitive material is insufficient in a low-temperature environment, resulting in a relatively low response value. As the temperature rises, more gas molecules can obtain enough energy to increase the diffusion and reaction speed, and the response value increases. However, as the test temperature rises, the gas molecules diffuse too quickly and desorb too fast, preventing them from reaching further, and the response value falls accordingly. Furthermore, adsorption and desorption are in a state of dynamic equilibrium. When the temperature is too high, the gas desorption rate may be greater than the adsorption rate, and the gas sensitivity response value will decrease. At the same time, the sensor devices based on TiO_2_−Ag and CoFe_2_O_4_−Ag are produced as a comparison. It is discovered that their resistance is excessively high; only when the temperature exceeds 360 °C does the resistance fall within the instrument’s test range. Furthermore, their response value is extremely low, less than 1.7 for 50 ppm toluene, and they do not exhibit a similar response value to TiO_2_−CoFe_2_O_4_−Ag with a significant temperature-related change trend. At the same time, their response value error is larger than that of TiO_2_−CoFe_2_O_4_−Ag. Finally, it is found that the best working temperature of TiO_2_−CoFe_2_O_4_−Ag is 263 °C, and the response value is about 15 for 50 ppm toluene gas. As a result, all subsequent performance tests are conducted using TiO_2_−CoFe_2_O_4_−Ag for toluene at 263 °C.

As shown in Figure 5c, it can be found that the response/recovery time generally shows a gradually increasing trend as the concentration of toluene gas increases. When the gas concentration is 3 ppm, the response/recovery time is 40 s/51 s.

The dynamic response test results in Figure 5d show that when the toluene concentration is between 3 ppm and 50 ppm, the response value increases linearly from 2 to 15, indicating that the synthesized TiO_2_−CoFe_2_O_4_−Ag gas-sensitive material can provide a reliable quantitative analysis of toluene. In addition, the limit of detection (LOD) can be estimated as 3δ/s based on the linear fitting, in which δ is the standard deviation and s is the slope of the linear fit. Finally, the LOD of TiO_2_−CoFe_2_O_4_−Ag to toluene is calculated to be about 10 ppb.

**Figure 6:**
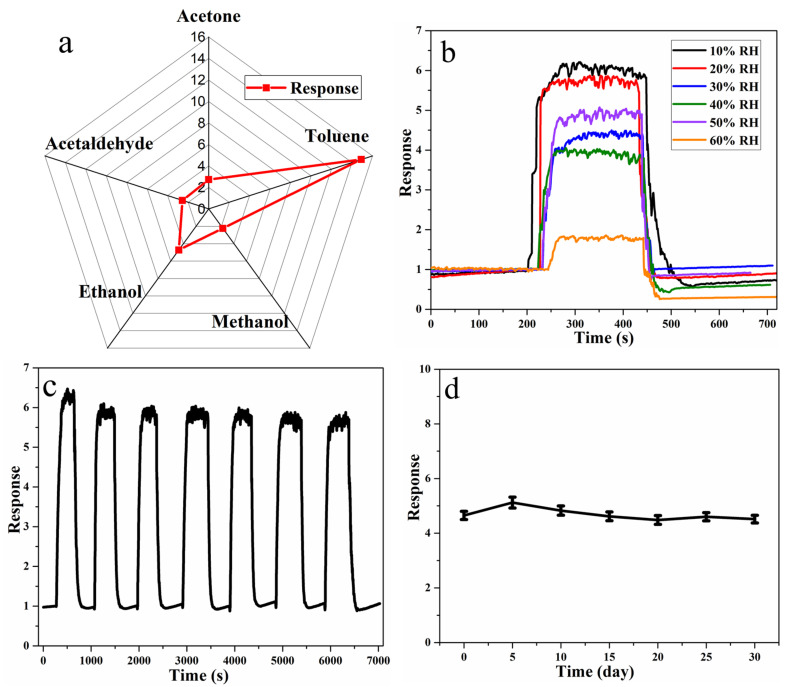
(**a**) Selectivity of five gases, (**b**) gas sensitivity test of toluene gas in different relative humidity (RH) environments, (**c**) repeatability and (**d**) long-term stability for toluene gas of TiO_2_−CoFe_2_O_4_−Ag at 263 °C (n = 3).

Then, the selectivity of the TiO_2_−CoFe_2_O_4_−Ag sensor to toluene gas is tested, using acetone, methanol, ethanol, and acetaldehyde as reference interference gases. Figure 6a depicts the gas sensitivity response of TiO_2_−CoFe_2_O_4_−Ag to different gases with the same concentration (50 ppm) at 263 °C, with the response values for each type of gas being approximately 3.5, 2.5, 4.5, 3, and 15, respectively. It can be observed that the TiO_2_−CoFe_2_O_4_−Ag-based sensor shows a much higher response to toluene than to other gases, indicating that it can accurately identify toluene from the above various interference gases.

The TiO_2_-CoFe_2_O_4_-Ag sensor is then tested for moisture resistance by placing it in different RH (10%–60% RH) conditions. Figure 6b shows that as the RH increases, the sensor response value to 20 ppm toluene decreases from about 6 to about 1.7. It is speculated that water vapor molecules in the environment occupy the surface of the gas-sensitive material, which affects the adsorption and reaction of oxygen molecules and target gases on the surface of the gas-sensitive material.

Following that, the sensor is tested for repeatability and long-term stability (Figure 6c, d), and it is discovered that the response value is stable at around 6, and the baseline does not drift significantly during the seven cycles of testing in the 20 ppm toluene atmosphere. In the long-term stability test, the response of TiO_2_−CoFe_2_O_4_−Ag to 10 ppm toluene gas was tested every 5 days; the result shows that about 90% of its response value is still maintained after 30 days.

**Figure 7:**
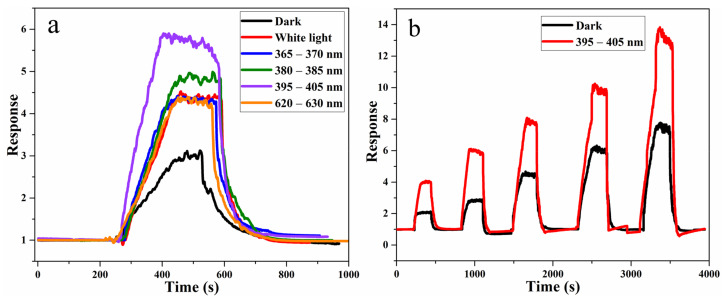
(**a**) Test of gas sensitivity of TiO_2_−CoFe_2_O_4_−Ag to toluene at 263 °C in darkness and under the light of different wavelengths, and (**b**) dynamic response curve of TiO_2_−CoFe_2_O_4_−Ag to toluene gas in darkness and under UV irradiation.

The light-excited gas sensitivity response test is then performed. As shown in Figure 7a, light irradiation with the same light intensity but different wavelengths have different enhancement effects on gas sensitivity. However, the gas sensitivity response value for 20 ppm toluene with light is higher than that in darkness. The response value of the device is the largest (about 6) with a wavelength of 395 nm–405 nm, which is much greater than that in darkness (about 3).

Finally, the dynamic response curve of TiO_2_−CoFe_2_O_4_−Ag with the light of 395 nm–405 nm wavelength and in darkness is displayed in Figure 7b. It is discovered that as the concentration of toluene gas increases within the measured concentration range (3 ppm–30 ppm), whether with or without light, the response value increases. The response value in the presence of light is much higher than that in darkness. This is mainly due to the effect of photogenerated electron–holes generated under light conditions on the gas-sensing performance.

### 3.3. Gas-Sensing Mechanism

When a metal oxide resistive semiconductor gas sensor comes into contact with gas molecules, the surface adsorption or reaction causes carrier movement, which causes changes in electrical conductivity, volt–ampere characteristics, or surface potential. Furthermore, various gases can be detected based on changes before and after exposure to the target gas [48,49,50]. At the same time, according to the literature, the gas sensitivity can be improved by surface modification, applying UV light illumination, and other methods [51,52].

**Figure 8:**
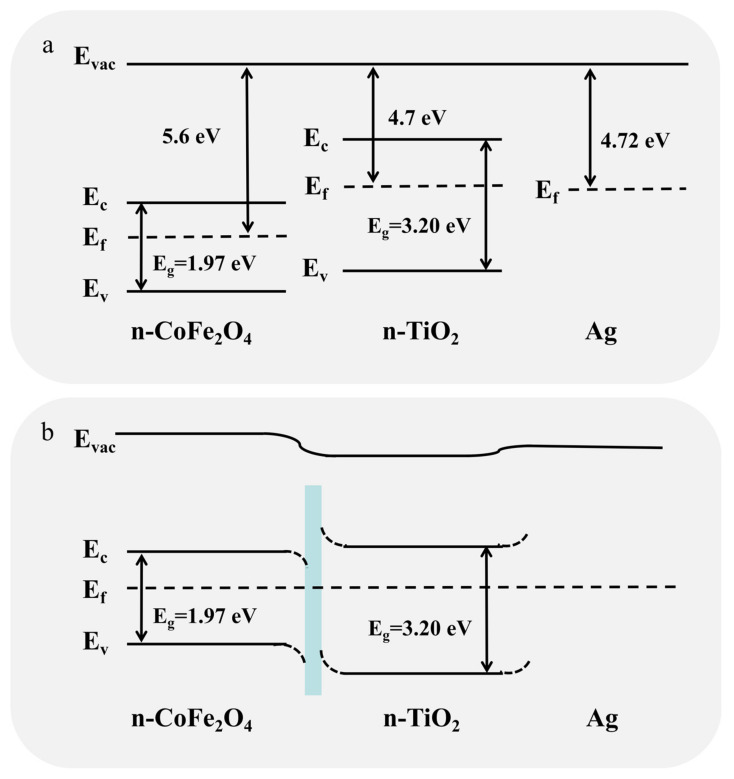
(**a**) Band structure diagram of TiO_2_, CoFe_2_O_4_, and Ag. (**b**) Band structure diagram of TiO_2_−CoFe_2_O_4_−Ag.

As illustrated in Figure 8a,b, when n-type TiO_2_ and n-type CoFe_2_O_4_ combine to form a heterojunction, electrons will flow from the side with the smaller work function to the side with the larger work function until the Fermi level reaches equilibrium [53,54,55]. An electron depletion layer will form on the TiO_2_ side, and the resistance in the air increases. After contacting the target gas, the change in resistance increases, and the sensitivity is improved. Furthermore, due to the current electronic effects, the formation of heterostructures between different metal oxide semiconductors can significantly improve O_2_ adsorption [55]. These effects work synergistically, which further improves the sensitivity of TiO_2_−CoFe_2_O_4_−Ag.

Moreover, the doped noble metal will form a Schottky junction with the gas-sensitive material [56]. More chemisorbed oxygen will be adsorbed as a result of the catalytic effect of the noble metals. Noble metal spillover allows chemisorbed oxygen species to be easily transported and distributed on the surface of gas-sensitive material, increasing the reaction rate [57]. In addition, chemical sensitization benefits from the catalytic properties of noble metals can reduce the surface reaction barrier, thereby lowering the working temperature and shortening the response time [58,59,60,61]. Furthermore, silver has good electrical conductivity, which promotes electron transfer and accelerates the ionization of oxygen and the surface redox reaction, ultimately improving response speed [62].

Furthermore, adding light irradiation will produce photogenerated carriers to further improve its gas sensitivity [63,64]. Light excitation can also provide energy for the transition of carriers between energy bands and can activate the surface to adsorb oxygen, resulting in increased sensitivity [42]. These are the reasons why adding light irradiation leads to the enhancement of the response intensity in this work.

At the same time, in addition to the chemical sensitization caused by noble metals, which can lower the working temperature, the composite cobalt ferrite has good catalytic activity and conductivity. Because cobalt ferrite is a multivalent metal element substance, the electron can jump in the cation valence of the octahedral position in its spinel structure [38,65]. The bandgap is significantly reduced after combining titanium dioxide, cobalt ferrite, and silver as shown in Figure 4d. As a result, electrons transition more easily, and the material’s conductivity increases. Eventually, this leads to a decrease in operating temperature from 360 °C to 263 °C.

Furthermore, during the sensing process, there may be some physical charge transfer between the toluene gas and the sensing layer, resulting in a decrease in resistance.

## 4. Conclusions

In summary, the TiO_2_−CoFe_2_O_4_−Ag quaternary composite gas-sensing material was prepared using the hydrothermal method. The detection capability of the heterojunction to toluene was significantly improved by doping noble metal, which could induce the electronic sensitization and chemical sensitization effects. The working temperature was reduced from 360 °C to 263 °C, and the response value to 50 ppm toluene was increased from 1.7 to 15. At the same time, TiO_2_−CoFe_2_O_4_−Ag has good selectivity and long-term stability. Furthermore, the response value of TiO_2_−CoFe_2_O_4_−Ag to UV irradiation was increased for toluene gas. This article introduces a novel approach to the development of an excellent gas-sensitive reaction system.

## Figures and Tables

**Table 1:** Physical properties of TiO_2_−Ag, CoFe_2_O_4_−Ag, and TiO_2_−CoFe_2_O_4_−Ag.

Sample	Specific Surface Area (m^2^/g)	Pore Volume (cm^3^/g)	Pore Diameter (nm)
TiO_2_−Ag	63.21	0.324	20.21
CoFe_2_O_4_−Ag	102.75	0.155	8.84
TiO_2_−CoFe_2_O_4_−Ag	59.04	0.304	19.67
